# Supplementary material for: Vitamin D Modified DSS-Induced Colitis in Mice via STING Signaling Pathway
Source: Biology (Basel). 2025 Jun 18;14(6):715. doi: 10.3390/biology14060715 (PMC12190092; doi:10.3390/biology14060715)
Supplement: Supplementary file 1 [file biology-14-00715-s001.zip › Supplementary Materials S1: 16s-report of gut microbiota/Groups/diff_analysis/heatmap/ECdiffheatmap.pdf]

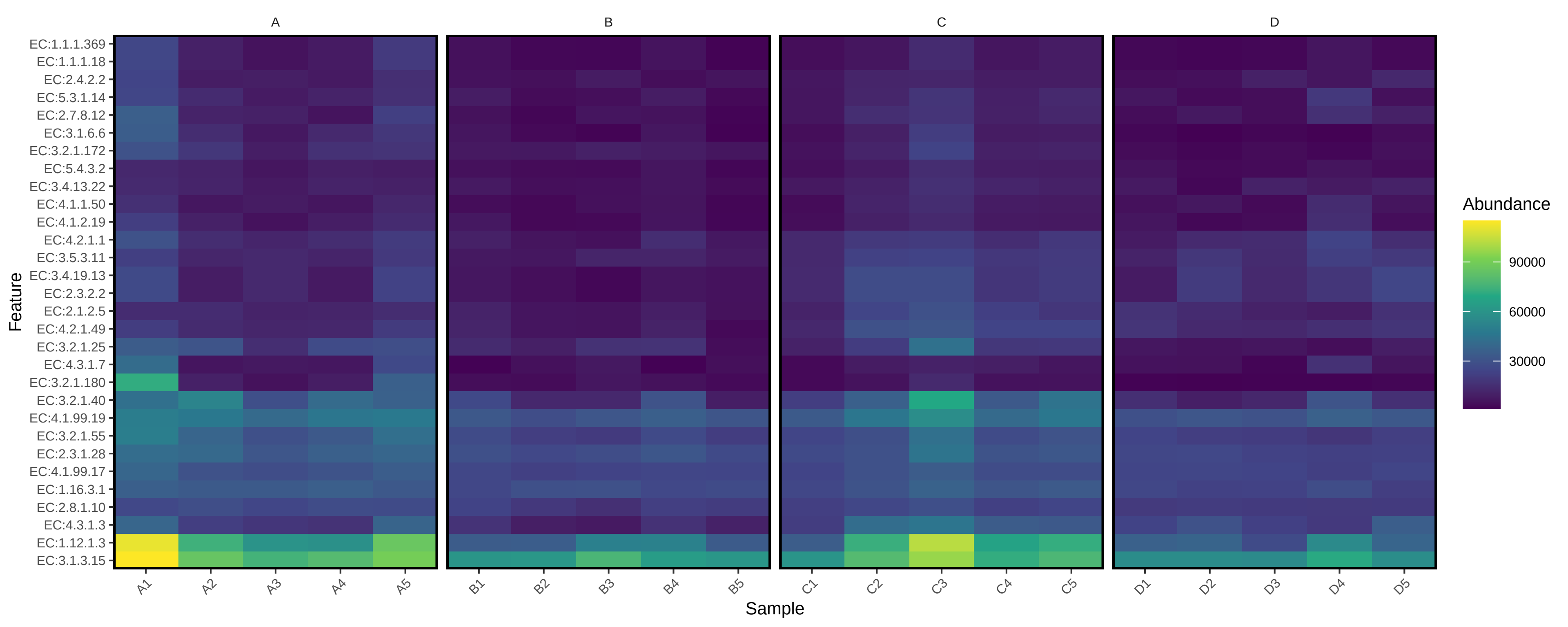
A
B
C
D
Feature
EC:1.1.1.369
EC:1.1.1.18
EC:2.4.2.2
EC:5.3.1.14
EC:2.7.8.12
EC:3.1.6.6
EC:3.2.1.172
EC:5.4.3.2
EC:3.4.13.22
EC:4.1.1.50
EC:4.1.2.19
EC:4.2.1.1
EC:3.5.3.11
EC:3.4.19.13
EC:2.3.2.2
EC:2.1.2.5
EC:4.2.1.49
EC:3.2.1.25
EC:4.3.1.7
EC:3.2.1.180
EC:3.2.1.40
EC:4.1.99.19
EC:3.2.1.55
EC:2.3.1.28
EC:4.1.99.17
EC:1.16.3.1
EC:2.8.1.10
EC:4.3.1.3
EC:1.12.1.3
EC:3.1.3.15
A1
A2
A3
A4
A5
B1
B2
B3
B4
B5
C1
C2
C3
C4
C5
D1
D2
D3
D4
D5
Sample
Abundance
90000
60000
30000
